# The role of cognitive reserve and clinical symptoms in the association between genetic liability for educational attainment and functioning in first-episode psychosis: A mediation analysis

**DOI:** 10.1192/j.eurpsy.2023.2480

**Published:** 2024-01-05

**Authors:** Derek Clougher, Àlex G. Segura, Maria F. Forte, Gisela Mezquida, Manuel J. Cuesta, Eduard Vieta, Silvia Amoretti, Antonio Lobo, Ana González-Pinto, Covadonga M. Díaz-Caneja, Alexandra Roldán, Giovanna Fico, Elena de la Serna, Daniel Bergé, Patricia Gassó, Natalia Rodriguez, Norma Verdolini, Alfonso Tortorella, Giulia Menculini, Marta Ribasés, Miguel Bernardo, Sergi Mas, PEPs Group

**Affiliations:** 1 Bipolar and Depressive Disorders Unit, Hospital Clinic of Barcelona, Institute of Neurosciences, IDIBAPS, University of Barcelona, Barcelona, Spain; 2 Biomedical Research Networking Center for Mental Health Network (CIBERSAM), Barcelona, Spain; 3 Department of Clinical Foundations, Pharmacology Unit, University of Barcelona, Barcelona, Spain; 4 Barcelona Clinic Schizophrenia Unit, Hospital Clinic of Barcelona, Neuroscience Institute, University of Barcelona, August Pi i Sunyer Biomedical Research Institute (IDIBAPS), Barcelona, Spain; 5 Department of Psychiatry, Complejo Hospitalario de Navarra, Pamplona, Spain; 6 IdiSNA, Navarra Institute for Health Research, Pamplona, Spain; 7 Psychiatric Genetics Unit, Group of Psychiatry, Mental Health and Addictions, Vall d’Hebron Research Institute (VHIR), Barcelona, Spain; 8 Department of Medicine and Psychiatry, Zaragoza University, Instituto de Investigación Sanitaria Aragón (IIS Aragón), Zaragoza, Spain; 9 Araba University Hospital, Bioaraba Research Institute, Vitoria-Gasteiz, Spain; 10 University of the Basque Country (UPV-EHU), Bilbao, Spain; 11 Department of Child and Adolescent Psychiatry, Institute of Psychiatry and Mental Health, Hospital General Universitario Gregorio Marañón, School of Medicine, Universidad Complutense, IiSGM, Madrid, Spain; 12 Department of Psychiatry, Institut d’Investigació Biomèdica-Sant Pau (IIB-SANT PAU), Hospital de la Santa Creu i Sant Pau, Universitat Autònoma de Barcelona (UAB), Barcelona, Spain; 13 Department of Child and Adolescent Psychiatry and Psychology, 2017SGR881, Institut Clinic de Neurociències, Hospital Clínic Universitari, IDIBAPS, Department of Medicine, University of Barcelona, Barcelona, Spain; 14 Hospital del Mar Medical Research Institute, MELIS Department, Universitat Pompeu Fabra, Barcelona, Spain; 15 Local Health Unit Umbria 1, Department of Mental Health, Mental Health Center of Perugia, Perugia, Italy; 16 Section of Psychiatry, Department of Medicine and Surgery, University of Perugia, Perugia, Italy; 17 Serra-Hunter Fellow, Department of Basic Clinal Practice, Pharmacology Unit, University of Barcelona.

**Keywords:** cognitive reserve, first-episode psychosis, functioning, negative symptoms, polygenic risk score

## Abstract

**Background:**

Polygenic risk scores for educational attainment (PRS_EA_), cognitive reserve (CR), and clinical symptoms are associated with functioning in first-episode psychosis (FEP). Nevertheless, the mechanisms underlying their complex interaction are yet to be explored. This study assessed the mediating role of CR and clinical symptoms, both negative (NS) and positive (PS), on the interrelationship between PRS_EA_ and functionality, one year after a FEP.

**Methods:**

A total of 162 FEP patients underwent clinical, functional, and genetic assessments. Using genome-wide association study summary results, PRS_EA_ were constructed for each individual. Two mediation models were performed. The parallel mediation model explored the relationship of PRS_EA_ with functionality through CR and clinical symptoms. The serial mediation model tested a causal chain of the three mediators: CR, NS, and PS. Mediation analysis was performed using the PROCESS function V.4.1 in SPSS V.22.

**Results:**

A serial mediation model revealed a causal chain for PRS_EA_ > CR > NS > Functionality (*β* = −0.35, 95%CI [−0.85, −0.04], *p* < 0.05). The model fit the data satisfactorily (CFI = 1.00; RMSEA = 0.00; SRMR = 7.2 × 10^−7^). Conversely, no parallel mediation was found between the three mediators, PRS_EA_ and functionality and the model poorly fit the data (CFI = 0.30; RMSEA = 0.25; SRMR = 0.11).

**Conclusions:**

Both CR and NS mediate the relationship between PRS_EA_ and functionality at one-year follow-up, using serial mediation analysis. This may be relevant for prevention and personalized early intervention to reduce illness impact and improve functional outcomes in FEP patients.

## Introduction

First-episode psychosis (FEP) is characterized by functional impairments in social, occupational, and independent living activities and is a crucial period for early intervention to improve long-term prognosis [1, 2]. Achieving functional remission in FEP is a core clinical objective [3], yet recovery rates vary over the course of the illness [4–6], with long-term functioning impairments present even in patients in clinical remission [7–9]. Several factors are believed to influence functioning in FEP patients, including genetic variability [2], negative symptoms [10–12], cognitive performance [13, 14], and cognitive reserve (CR) [15, 16].

Genetic variability is a potential modulator of prognosis in FEP [2] and is understood using polygenic risk scores (PRSs) [17]. PRSs aggregate the effects of many genetic variants across the human genome into a single score and are used to predict the genetic disposition for developing a given disease, including mental disorders [18], while also overcoming certain limitations of candidate-gene strategies [2]. In fact, PRSs demonstrated good discriminative ability of case–control status in FEP individuals [2, 19]. Schizophrenia and bipolar disorder PRSs have been linked to symptom severity, comorbid disorders, and cognitive impairments [20]. A significant positive correlation between PRS and the Positive and Negative Syndrome Scale (PANSS) but not overall functioning was found in a sample of FEP individuals [21]. Another study [22] failed to find an association between schizophrenia PRSs and functioning. In both studies, the inclusion of patients at different illness stages with varying symptomatology and small sample sizes may have reduced power to identify small effects. The PRS for educational attainment (PRS_EA_) is based on the completed years of schooling and captures associated social, economic, and health outcomes [23]. Lower educational attainment is associated with higher schizophrenia PRSs [24] and an overall higher frequency of copy number variants (CNVs) which are considered high risk for psychiatric disorders [25]. Importantly, a higher PRS_EA_ was associated with lower symptom severity and better functionality suggesting increased autonomy and better cognitive functioning [2], thus highlighting the potential protective properties of PRS_EA_.

CR has also been considered a protective factor and is understood as the brain’s ability to cope in response to pathology and delay the onset of the associated clinical, cognitive, and functional symptoms [26–30]. In various psychiatric populations, including FEP, higher CR has been associated with later onset age, greater insight, and reduced illness severity in terms of symptoms, particularly negative symptoms, better cognitive performance, and functioning [15, 31–34]. Individual differences in CR could explain why people with similar disorders differ in their levels of functioning [30, 32, 34–36].

Research exploring PRSs and their associations with CR, clinical symptoms, and functioning following a FEP remains limited. Understanding the factors contributing to functional performance in FEP may contribute to early personalized intervention and person-focused therapy. The aim of this study was to investigate the mediating role of CR and clinical symptoms (negative and positive) on the interrelationship between genetic liability for educational attainment and functionality one-year post-FEP. We hypothesize that patients with higher PRS_EA_ will have higher CR and less clinical symptoms, thus better overall functionality at one-year follow-up.

## Methods

### Sample

A total of 335 FEP patients participated in the “Phenotype–Genotype Interaction: Application of a Predictive Model in First Psychotic Episodes” (PEPs based on Spanish acronym) [37, 38], a collaborative project between various members of the Spanish Research Network on Mental Health (CIBERSAM) [39]. This was a multicentre, naturalistic, prospective, longitudinal study. For comprehensive information regarding medication and sample diagnosis see Bioque et al. [40].

The PEPS study inclusion criteria were: (1) between 7 and 35 years at first evaluation; (2) < 12 months history of psychotic symptoms; (3) fluent Spanish, and (4) provide written informed consent. Exclusion criteria were: (1) intellectual disability according to DSM-IV-TR criteria; (2) history of head trauma with loss of consciousness, and (3) organic disease with mental repercussions.

Patients who provided blood samples for genetic analysis, passed the genetic quality control (see section: blood samples and genotyping), completed all assessments at one-year follow-up, were aged ⩾16 years old (chosen cut-off point as this is the age at which most scales report adolescent-adulthood results), had self-reported European ancestry, belonged to the non-affective psychotic disorder diagnostic category and, additionally, had all the information needed to calculate CR, were included. To control for the potential loss of sample, we focused on symptomatology and functional data for a period of 1 year. Supplementary Figure 1 depicts the selection process of the 162 patients with FEP.

The PEPs Project was approved by the Clinical Research Ethics Committee of all participating centers and was conducted in accordance with the ethical principles of the Declaration of Helsinki and Good Clinical Practice.

Written informed consent was obtained from all participants prior to inclusion in the study.

## Assessments

### Clinical, pharmacological, and sociodemographic assessment

Relevant sociodemographic, clinical, and pharmacological data were collected for all participants. Sociodemographic data included age, sex, and education. Pharmacological treatment was based on international consensus [41] and measured using chlorpromazine equivalents (CPZ). To calculate the duration of untreated psychosis (DUP), the number of days between the time taken from the initial onset of psychotic symptoms to beginning treatment for psychosis was calculated. The onset of psychotic symptoms was assessed with the Symptom Onset in Schizophrenia (SOS) scale [37, 42], explored via interviews with the patient, medical records, and interviews with relatives.

Diagnoses were established using the Structured Clinical Interview for DSM (SCID-I-II) [43, 44] according to DSM-IV criteria. The PANSS scale [45] was administered for the psychopathology assessment. Higher scores indicate greater symptom severity.

Although the PANSS is one of the most widely used measures of negative symptom severity, it has several limitations as it was not designed to evaluate negative symptoms exclusively [46]. Thus, we also used the PANSS-Marder Factor Scores [47] as it has more restrictive criteria to assess positive and negative symptomatology. For the present study, the PANSS was solely used to understand the role of positive and negative symptoms in the sample as the literature has shown that CR is highly associated with negative symptoms only [31], whereas functionality has been linked to both positive and negative symptoms [48]. The sum of the following items of the PANSS were used to calculate the Positive Symptom Factor (PS): delusions (P1), hallucinatory behavior (P3), grandiosity (P5), suspiciousness/persecution (P6), stereotyped thinking (N7), somatic concerns (G1), unusual thought content (G9) and lack of judgment and insight (G12); and for the Negative Symptom Factor (NS): blunted affect (N1), emotional withdrawal (N2), poor rapport (N3), passive/apathetic social withdrawal (N4), lack of spontaneity and conversation flow (N6), motor retardation (G7) and active social avoidance (G16).

### Functional assessment

The Functioning Assessment Short Test (FAST) [49] evaluated overall functioning across the following six areas: autonomy, occupational functioning, cognitive functioning, management of personal finances, interpersonal relationships, and leisure time. Higher scores indicate poorer functioning.

The Premorbid Adjustment Scale (PAS) [50] evaluates the achievement of developmental goals prior to the onset of psychotic symptoms and was administered retrospectively to assess premorbid adjustment. Information was obtained from the patients themselves and parents/close relatives. All participants completed the childhood and adolescence elements of this scale. Higher scores indicate worse premorbid adjustment.

### CR assessment

Premorbid intelligence quotient (IQ), educational attainment level, and lifetime participation in leisure, social, and physical activities are the three most commonly proposed proxy indicators of CR in psychiatry, particularly in FEP [31–33, 35, 36] and were used to assess CR in this study. Estimated premorbid IQ was evaluated with the Vocabulary subtest of the Wechsler Adult Intelligence Scale-III [51] as a measure of crystallized intelligence. The total number of participants’ completed years in education, as well as parents’ educational level, were used to assess educational attainment level. The scholastic performance domain of the PAS scale was used to evaluate lifetime participation in leisure, social, and physical activities and by enquiring about involvement in social activities, their self-rated capacity to take part in physical activities and satisfaction with hobbies. Higher scores indicate better performance. A “Cognitive Reserve Score” was created via a Principal Components Analysis (PCA) for each subject with completed data for the three core proxy indicators.

### Blood samples and genotyping

K2EDTA BD Vacutainer EDTA tubes (Becton Dickinson, Franklin Lakes, NJ) were used to collect blood samples, which were subsequently stored at −20°C prior to shipment to the central laboratory for further analysis. The MagNA Pure LC DNA isolation kit – large volume and MagNA Pure LC 2.0 Instrument (Roche Diagnostics GmbH, Mannheim, Germany) supported DNA extraction and DNA concentration was determined by absorbance (ND1000, NanoDrop, Wilmington, Delaware). Specifically, 2.5 μg of genomic DNA was sent for genotyping at the Spanish National Genotyping Centre (CeGen) using Axiom Spain Biobank Array (developed in the University of Santiago de Compostela, Spain).

### PRS calculation

Genotyping data were submitted to the Michigan Imputation Server [52], following the standard pipeline for Minimac4 software and setting a European population reference from build GRCh37/hg19, reference panel HRC 1.1 2016 and Eagle v2.4 phasing.

For PRS calculation, genome-wide association study (GWAS) summary results from the Social Science Genetic Association Consortium were obtained. Based on our previous study [2] we selected the PRS_EA_ (1,131,881 individuals) [23], measured as the number of years of schooling that individuals completed. Higher scores reflect the genetic liability for higher educational attainment. Duplicated and unknown strand GWAS summary single-nucleotide polymorphisms (SNPs) were excluded.

Quality control was performed with PLINK v1.07 [53]. Inclusion criteria for SNPs were minor allele frequency > 0.01, Hardy–Weinberg equilibrium *p* > 10^−6^, marker missingness <0.01, and imputation INFO >0.8. Pruning was done using a window/step size of 200/50 kb and *r* ^2^ > 0.25. Sample quality control included individuals with heterozygosity values within three standard deviations (SD) from the mean, a missingness rate of <0.01, matching chromosomal and database-labeled sex and relatedness π-hat <0.125.

The PRS were constructed using PRS-CS, a method that implements a high-dimensional Bayesian regression to perform a continuous shrinkage of SNP effect sizes using GWAS summary statistics and an external linkage disequilibrium (LD) reference panel [54]. The LD reference panel was constructed using a European subsample of the UK Biobank [55]. For the remaining parameters, the default options as implemented in PRS-CS were adopted.

A genetic principal component analysis (PCA) was performed to control population stratification [56] by means of the SNPRelate package, and the first 10 components were used as covariates in the statistical analyses including PRS.

## Statistical analysis

The normality of continuous variables was tested using the Kolmogorov–Smirnov and Shapiro–Wilk tests. The confounding effect on functionality of discrete variables was analyzed using a *t*-test and Pearson’s correlation coefficient was used for continuous variables. Before testing the mediation hypothesis, we tested the relationship between mediators and the outcome variable using Pearson’s correlation coefficient.

Mediation analysis tested whether the effect of a causal variable (PRS_EA_) on an outcome variable (functionality, FAST scale score) is affected by one or more mediator variables (CR, NS, PS, and Marder PANSS Factor Scores) at one-year follow-up. The relationship between variables is described by three effects: (1) Total effect (*c*), the association between causal variable and outcome variable; (2) Direct effect (*c*’), the effect of the causal variable on the outcome variable, when controlling for the mediator variables; and (3) Indirect effect, the effect of the causal variable on the outcome variable via the mediator variable [57]. Two mediation models were explored. A parallel mediation model explored the relationship of PRS_EA_ with functionality through CR, NS, and PS. A serial mediation model tested a causal chain of the three mediators: CR, NS, and PS. Based on clinical knowledge, we propose that genetic predisposition for educational attainment may be linked to higher CR, which in turn decreases clinical symptomatology and therefore increases functionality (PRS_EA_ > CR > Clinical symptoms > Functionality) [2, 10, 32]. For each model, we obtained the total effect, the direct effect, and the total indirect effect of all mediator variables, as well as the indirect effect of each individual mediator or serial path.

The statistical significance of the indirect effect was tested with a nonparametric bootstrapping approach (5000 iterations) to obtain 95% confidence intervals. In these analyses, mediation is considered significant if the 95% bias-corrected for the indirect effect does not include 0.

Analysis was performed using the PROCESS function V.4.1 in SPSS V.22. The model 4 (model as a parameter in the PROCESS function) was used for the parallel mediation model, and model 6 for the serial mediation models. To control for population stratification, all models were fitted by the first 10 principal components of the PCA analysis. Model fit statistics were also reported using the following: a Comparative Fit Index (CFI) (satisfactory > 0.90), a Root Mean Square Error of Approximation (RMSEA) (satisfactory < 0.05), and a Standardized Root Mean Square Residual (SRMR) (satisfactory < 0.08) [58]. The fit indices were derived using the R package *lavaan* [59].

## Results

Table 1 shows the characteristics of the sample with 70% male and a mean age of 24.7 (SD = 5.4). The mean dose of antipsychotic medication was equivalent to 577.8 (SD = 489.5) mg/day of CPZ, and the mean DUP was 98.7 (SD = 128.2) days (14 weeks approximately).

**Table 1.** Main sociodemographic, functional, and clinical features of the FEP sample at study entry (*N* = 162)

Sociodemographic variables (Mean ± SD or *n* (%))	
Sex (Male/Female)	113(70)/49(30)
Age (years)	24.7 ± 5.4
Age at onset (years)	24.6 ± 5.4
Duration of untreated psychosis (days)	98.7 ± 128.2
Educational level	
No education	1 (0.6)
Primary education	27 (16.7)
Lower secondary education	63 (38.9)
Upper secondary and non-tertiary education	41 (25.3)
University	29 (17.9)
Others	1 (0.6)
Chlorpromazine equivalents	577.8 ± 489.5
Cannabis (yes)	73(44)
Tobacco (yes)	116(69)
Clinical and functional variables at baseline (Mean ± SD)	
Positive Marder PANSS Factor	20.8 ± 8.4
Negative Marder PANSS Factor	18.2 ± 8.0
Functionality (FAST)	27.7 ± 16.3
Cognitive Reserve	75.9 ± 11.8
Clinical and functional variables at one-year follow-up (Mean ± SD)	
Positive Marder PANSS Factor	12.4 ± 5.3
Negative Marder PANSS Factor	14.1 ± 6.4
Functionality (FAST)	18.2 ± 14.9

Abbreviations: FAST, Functioning Assessment Short Test; PANSS, Positive and Negative Syndrome Scale.

Functionality, measured using the FAST total score, was negatively correlated with PRS_EA_ (*r* = −0.21, *p* = 0.004) and CR (*r* = −0.23, *p* = 0.003), and positively correlated with NS (*r* = 0.69, *p* < 0.001) and PS (*r* = 0.56, *p* < 0.001), indicating that higher PRS_EA_ and CR are associated with better functional outcome. In contrast, higher levels of NS and PS are correlated with worse functional outcome. As these correlations were significant, the conditions required to perform mediation analysis were fulfilled.

**Figure 1.**
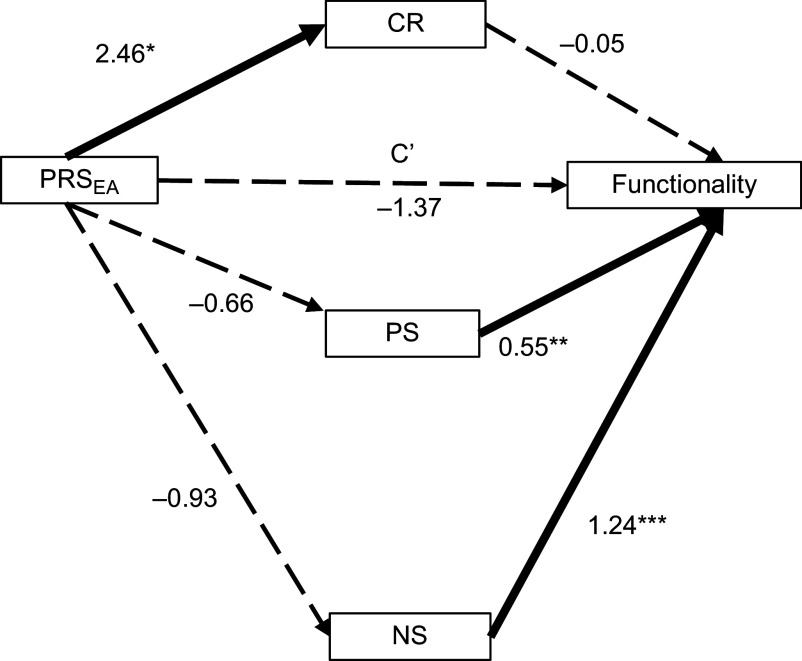
Parallel mediation model. The mediating effect of three mediators (CR, PS, and NS) in the relationship between PRS_EA_ and functionality. All presented effects are unstandardized. C′ is the direct effect of PRS_EA_ on functionality. *p < 0.05, **p < 0.01, ***p < 0.001. Continuous lines denoted significant regression. CR, cognitive reserve; NS, negative symptoms; PRS_EA_, polygenic risk score for educational attainment; PS, positive symptoms.

The total effect of PRS_EA_ on functionality was significant (*β* = −3.27, 95%CI [−5.62, −0.93], *p* = 0.006). In the parallel mediation model (Figure 1), the direct effect was not significant (*p* = 0.077), and a total indirect effect was present (*β* = −1.74, 95%CI [−3.27, −0.18], *p* < 0.05) (Table 2). None of the three mediators significantly mediated the relationship between PRS_EA_ and functionality. Fitting indices also demonstrated that the model poorly fit the data (CFI = 0.30; RMSEA = 0.25; SRMR = 0.11).

**Table 2.** Non-standardized total, direct, and indirect effects (total and of each individual mediator or path) of the two mediation models

	*β*	[95% CI]	*p*-value
1. Parallel mediation model			
Total effect	−3.08	[−5.34, −0.74]	**0.008**
Direct effect	−1.37	[−3.22, 0.48]	0.145
Total indirect effect	−1.71	[−3.35, −0.07]	**<0.05**
CR indirect effect	−0.13	[−0.59, 0.22]	>0.05
PS indirect effect	−0.7	[−0.97, 0.12]	>0.05
NS indirect effect	−1.21	[−2.77, 0.13]	>0.05
2. Serial mediation model			
Total effect	−3.08	[−5.34, −0.74]	**0.008**
Direct effect	−1.37	[−3.22, 0.48]	0.145
Total Indirect Effect	−1.71	[−3.35, −0.07]	**<0.05**
PRS_EA_ > CR > Functionality	−0.13	[−0.59, 0.23]	>0.05
PRS_EA_ > PS > Functionality	−0.09	[−0.57, 0.51]	>0.05
PRS_EA_ > NS > Functionality	−0.86	[−2.42, 0.52]	>0.05
PRS_EA_ > CR > PS > Functionality	−0.08	[−0.24, 0.01]	>0.05
PRS_EA_ > CR > NS > Functionality	−0.35	[−0.85, −0.04]	**<0.05**
PRS_EA_ > NS > PS > Functionality	−0.18	[−0.55, 0.14]	>0.05
PRS_EA_ > CR > NS > PS > Functionality	−0.07	[−0.21, 0.00]	>0.05

Abbreviations: CI, confidence interval; CR, cognitive reserve; NS, negative symptoms; PRS_EA_, polygenic risk score for educational attainment; PS, positive symptoms. Significant differences (p<0.05) marked in bold.

**Figure 2.**
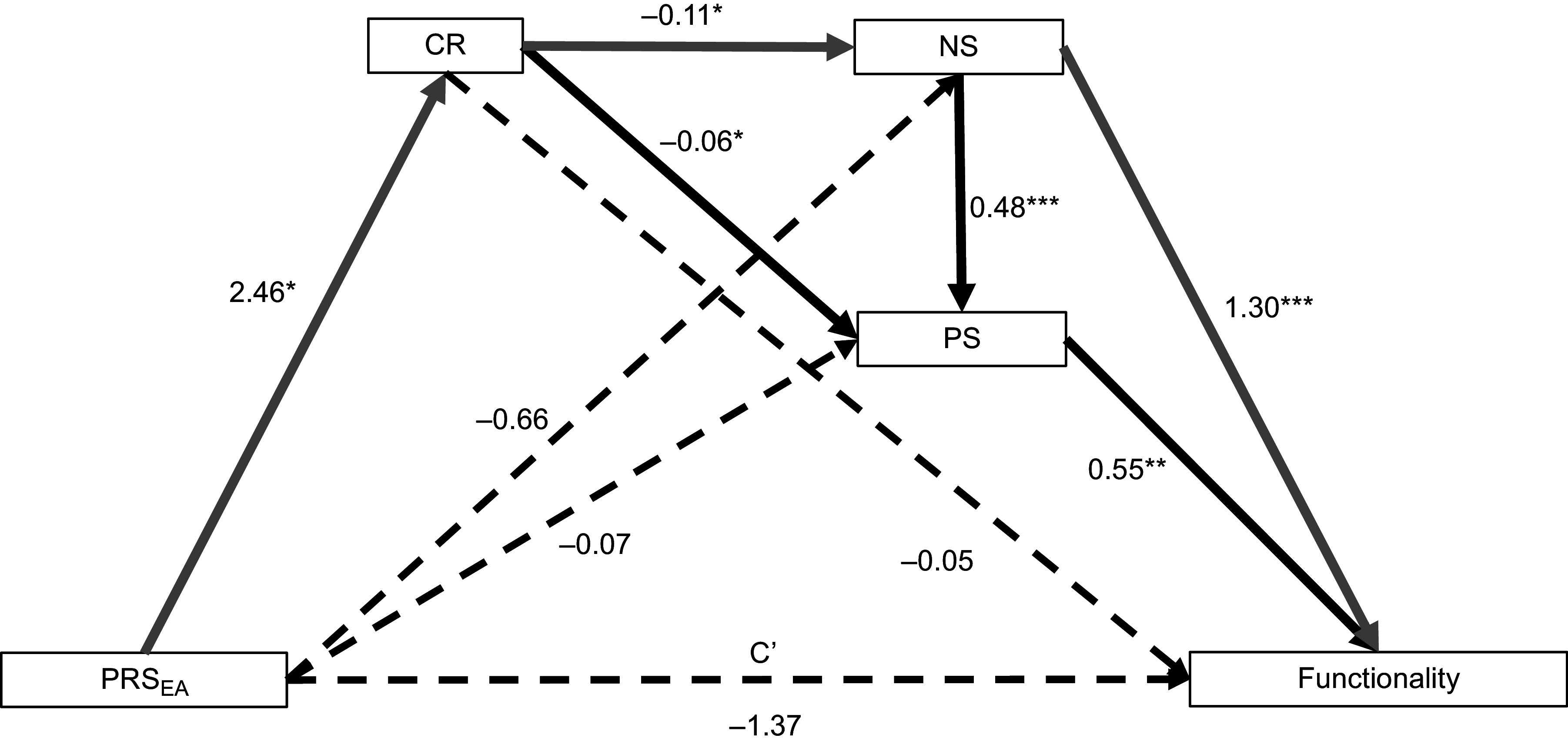
The serial mediating effect of CR, PS, and NS in the relationship between PRS_EA_ and functionality. All presented effects are unstandardized. C′ is the direct effect of PRS_EA_ on functionality. *p < 0.05, **p < 0.01, ***p < 0.001. Continuous lines denoted significant regression. Gray lines represent path with significant indirect effect. CR, cognitive reserve; NS, negative symptoms; PRS_EA_, polygenic risk score for educational attainment; PS, positive symptoms.

The serial mediation model hypothesizes a causal chain linking the three mediators in a specified order and direction flow. We propose that PRS_EA_ may be linked to higher CR, which in turn decreases clinical symptomatology and therefore increases functionality (Figure 2). Results show that the three mediators in the abovementioned causal order fully mediate the relationship between PRS_EA_ and functionality, as no direct effect was observed whereas the total indirect effect was significant (Table 2). Among the seven paths that could be inferred from the model, only the path including CR and NS as mediators was significant according to 5000 bootstrapped samples. Fitting indices indicated that the model fits the data satisfactorily (CFI = 1.00; RMSEA = 0.00; SRMR = 7.2 × 10^−7^).

## Discussion

The main finding of this study is that the serial mediation model demonstrated that CR and clinical symptoms, more specifically NS, mediate the relationship between PRS_EA_ and functionality at one-year follow-up. To the best of our knowledge, this is the first statistical model describing this causal chain of events, improving our understanding of previously observed clinical findings. Based on this causal relationship between variables, a parallel mediation model poorly fit the data. Our results provide evidence for the role of genetic liability in the cognitive and clinical aspects of FEP, further supporting findings for the association between PRS_EA_ (and not psychological PRSs) and cognition, illness course, and functioning [2]. In our serial model, a causal chain (PRS_EA_ > CR > Clinical symptoms > Functionality) was found.

In terms of CR, results indicate its potential genetic component. Genetic and environmental factors are both important in CR. Genetics determine individual aspects of functional brain processes, which can be influenced by the interaction of innate individual factors (e.g., in utero or genetically determined) as well as lifetime exposures. Conversely, environmental elements such as education, occupation, physical exercise, leisure activities, and social interaction are also influential [30, 60, 61]. In this context, the protective effect of the genetics underlying cognitive features in the early progression of clinical manifestation after a FEP has been recently reported [2]. As such, FEP individuals with an increased genetic predisposition for better cognitive functioning could be more resilient to the stressful effects of the psychotic episode and have a better prognosis [2]. Equally, environmental factors are currently addressed in specific interventions enhancing CR in FEP and high-risk populations [62]. Therefore, our results add to the previous research demonstrating the mediating effects of CR, while also including the genetic component and its influence in the relationship with clinical symptoms and functioning.

Regarding clinical aspects, different studies have shown that CR is closely linked with negative symptoms [31, 33] and only one study [48] has found a relationship between CR and positive symptomatology; the authors described that CR partially mediates the relationship between positive symptoms and functioning. Notably, negative and cognitive symptoms are indeed the primary predictors of functioning at different stages of psychotic disorders [63, 64], and appear to have a greater impact on functioning than positive symptoms [65, 66]. Several studies of the PEPs project have established the role of CR as a mediator of clinical and cognitive symptoms, as well as functionality [31, 32, 35, 67]. Amoretti and Ramos-Quiroga [15] found that higher levels of CR predict a better prognosis following a FEP, reiterating the need to consider the genetic component of this disorder. Nevertheless, in this study, CR alone did not predict functionality. This may be due to its complex interplay with NS and PS which are believed to have a more direct impact upon functionality [10]. Hence, CR may have a strong genetic basis which influences NS, PS, and functionality. Specifically, findings suggest that PRS_EA_ may lead to higher CR which in turn is linked to lower NS and therefore better functionality following a FEP (e.g., PRS_EA_ > CR > NS > Functionality). This finding is relevant for the application of PRS in personalized medicine which aims to improve early disease detection, as well as early prevention [68] and personalized intervention methods [69].

Certain limitations in the present study must be considered. Firstly, several constraints are associated with the use of the PANSS as it was not designed with the purpose of solely measuring negative symptoms [70]. To account for this, we used the PANSS-Marder Factor Scores [46] which applies stricter criteria for assessing positive and negative symptomatology. Future studies may include specific scales to assess negative symptoms such as the Brief Negative Symptom Scale (BNSS) [71, 72] to address this drawback. A similar limitation is seen in all studies measuring CR in psychiatric populations as at the time of conducting this study there were no validated tools to evaluate CR. The Cognitive Reserve Assessment Scale in Health (CRASH) [73] for adult population, and Cognitive Reserve Questionnaire for Adolescents (CoRe-A) [74] have since been designed and should be administered accordingly. Secondly, the limited sample size may increase the risk of reducing statistical power and the ability to detect small effects. As such, further research with larger sample sizes is required. Finally, the short follow-up period is a potential limitation in this study. Nonetheless, the present study is a naturalistic and multicentric study from the entire Spanish population and comprises the largest and best-characterized first-episode sample of the country. Additionally, the PRSs were calculated with the largest GWAS from international consortiums ergo the genetic variants have a greater capacity to capture the genetic susceptibility of the phenotypes explored. Furthermore, the specific PRS-CS method implemented ensures that the shrinkage of variant effect sizes allows the inclusion of all available SNPs in the PRSs and therefore avoids *p*-value thresholding.

## Conclusions

This study provides a potential clinical explanation for the association between genetic predisposition for educational attainment and functional outcomes. We identified an influence of CR on NS in mediating the relationship between PRS_EA_ and functioning in individuals with FEP. These results highlight the suitability and applicability of mediation models to explore the relationship between genetic and clinical data. Additionally, these results may be of significant clinical importance for two primary reasons. Firstly, we provide a clinical framework for clinicians by identifying a potential causal chain of events which can be part of the ongoing development of PRSs in precision psychiatry to further advance toward personalized interventions. Secondly, and based on these insights, the use of cognitive interventions could be recommended to enhance CR by focusing on mental stimulation (e.g., cognitive tasks), physical exercise, leisure activities, and social skills training [62, 75]. This is clinically relevant given the importance of functional outcomes during the first years after a first-episode. To prevent severe forms of the disorder and a poorer prognosis, rapid identification, timing of treatment, and early interventions in first episode patients are key factors in determining their prognoses and functional outcomes.

## Supporting information

Clougher et al. supplementary material Clougher et al. supplementary material

## Data Availability

The data that support the findings of this study are available on request from the corresponding authors.

## Supplementary material

The supplementary material for this article can be found at https://doi.org/10.1192/j.eurpsy.2023.2480.
