# Supplementary material for: The role of cognitive reserve and clinical symptoms in the association between genetic liability for educational attainment and functioning in first-episode psychosis: A mediation analysis
Source: Eur Psychiatry. 2024 Jan 5;68(1):e7. doi: 10.1192/j.eurpsy.2023.2480 (PMC11795430; doi:10.1192/j.eurpsy.2023.2480)
Supplement: Clougher et al. supplementary material [file S092493382302480Xsup001.docx]

**Supplementary Figure 1.** Flowchart detailing the selection process of the 166 patients with FEP included in this study

335 patients with a First Episode of Psychosis were recrutied in the PEPs Project

27 patients did not provide biological sample or failed genetic quality control

51 patients did not provide European Ancestry

65 patients did not have one year follow-up data

25 patients did not have cognitive data

162 patients with a First Episode of Psychosis were included in the present study

5 Patients aged<16 years old
